# Supplementary figures and images for: Hi-C implementation of genome structure for in silico models of radiation-induced DNA damage
Source: PLoS Comput Biol. 2020 Dec 16;16(12):e1008476. doi: 10.1371/journal.pcbi.1008476 (PMC7773326; doi:10.1371/journal.pcbi.1008476)

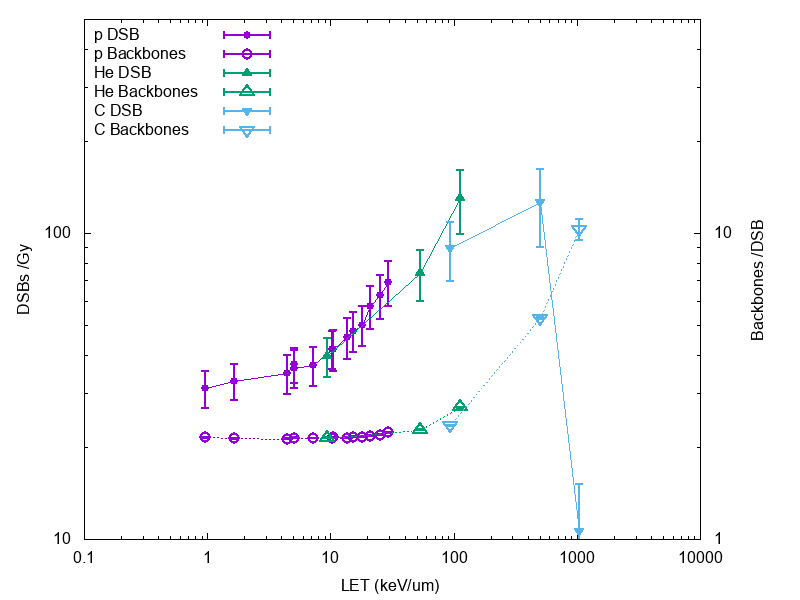

Supplement: S1 Fig — Average number of DSBs/Gy (filled symbols) and backbones per DSBs (empty symbols) for a range of LET values across different particle types. Error bars are displayed as the standard error of the mean for 100 repeats. (TIF) [file pcbi.1008476.s001.tif]

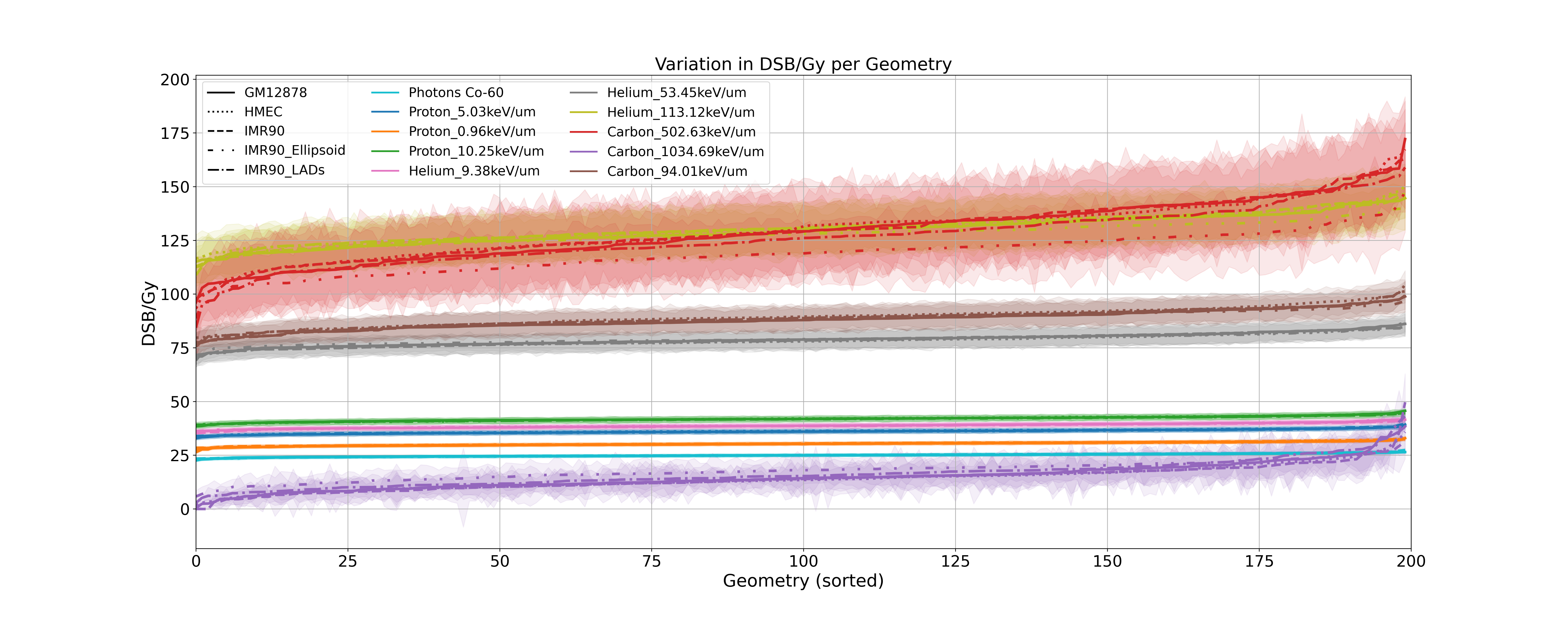

Supplement: S2 Fig — Yields of DSB per Gy of dose for the each of the 200 geometries created. Results have been sorted from smallest to largest yields to allow for easier interpretation. Different cell-types are shown as different line types with each radiation quality presented as a different colour. Errors are the transparent area around the line and are the standard error in the mean for 50 independent exposures per geometry. (TIF) [file pcbi.1008476.s002.tif]

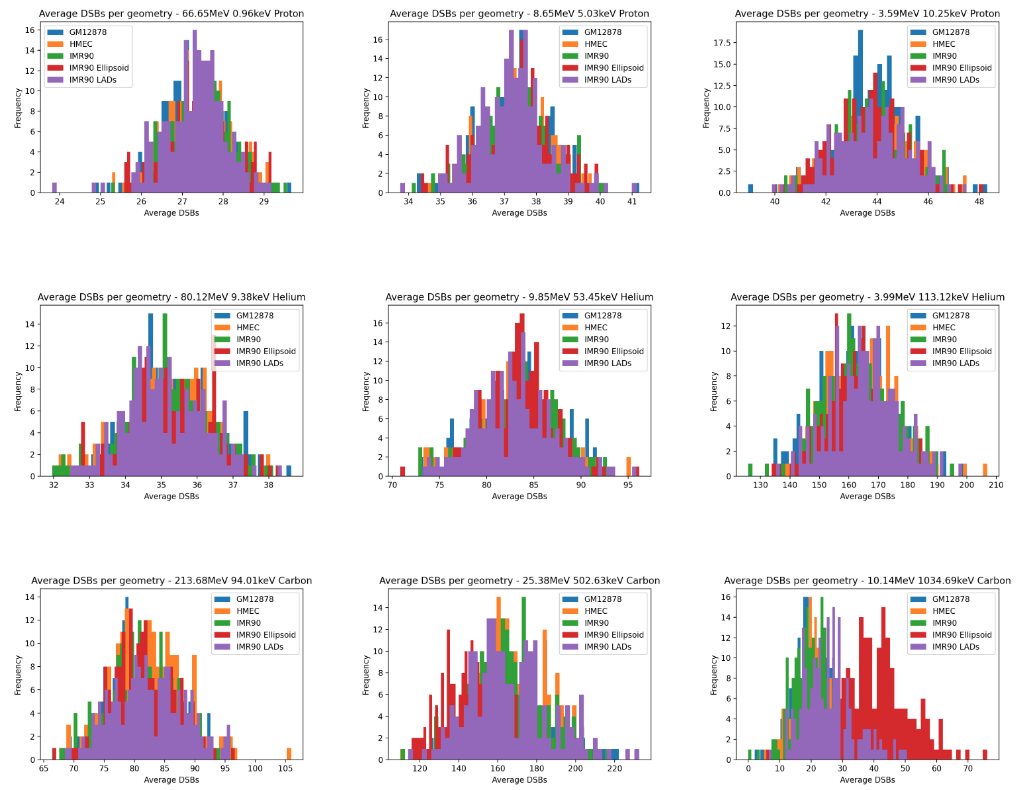

Supplement: S3 Fig — Double-strand break yield histograms for 200 geometries of each cell-type and variant. (TIF) [file pcbi.1008476.s003.tif]

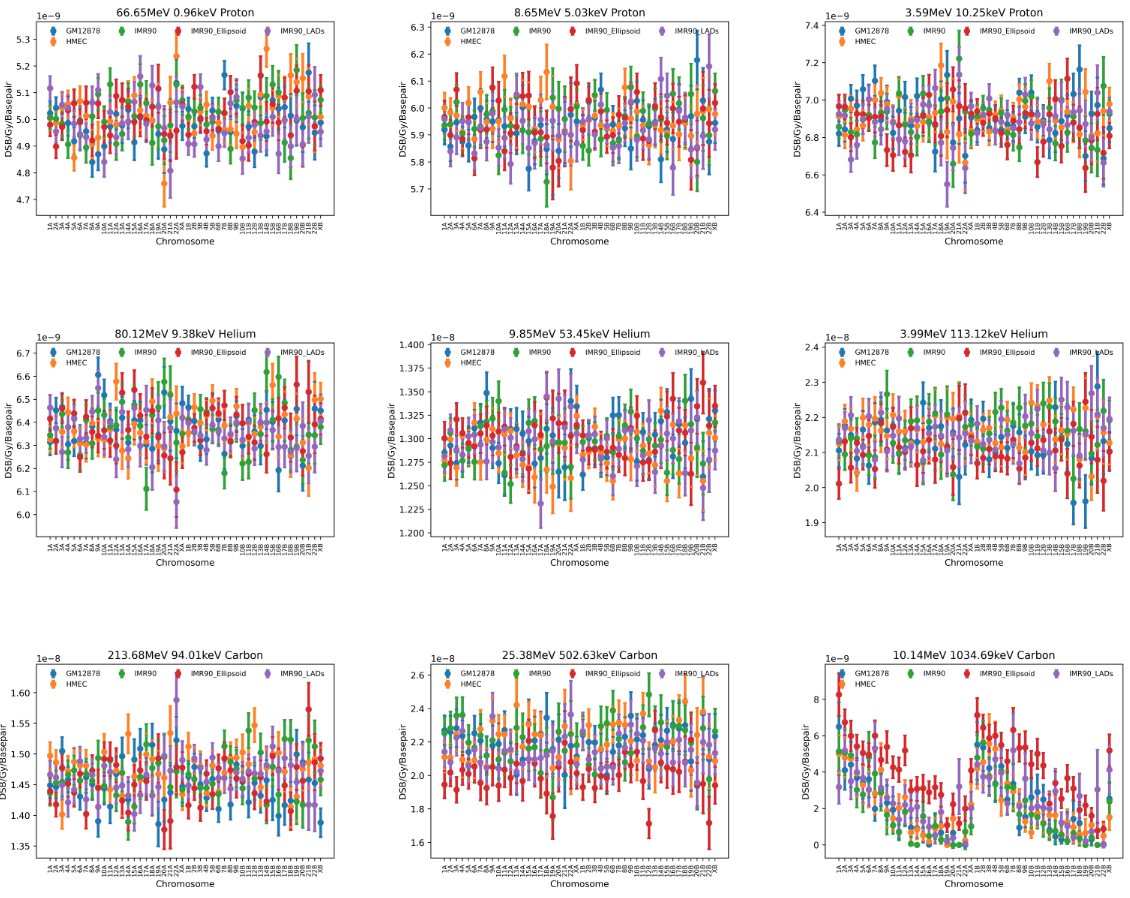

Supplement: S4 Fig — Double-strand break per 1Gy of dose per DNA basepair on each of the modelled 46 chromosomes for each cell-type and variant. Error bars are displayed as the standard error of the mean for 200 geometries for each cell-type and variant with each geometry having 50 independent exposures. (TIF) [file pcbi.1008476.s004.tif]

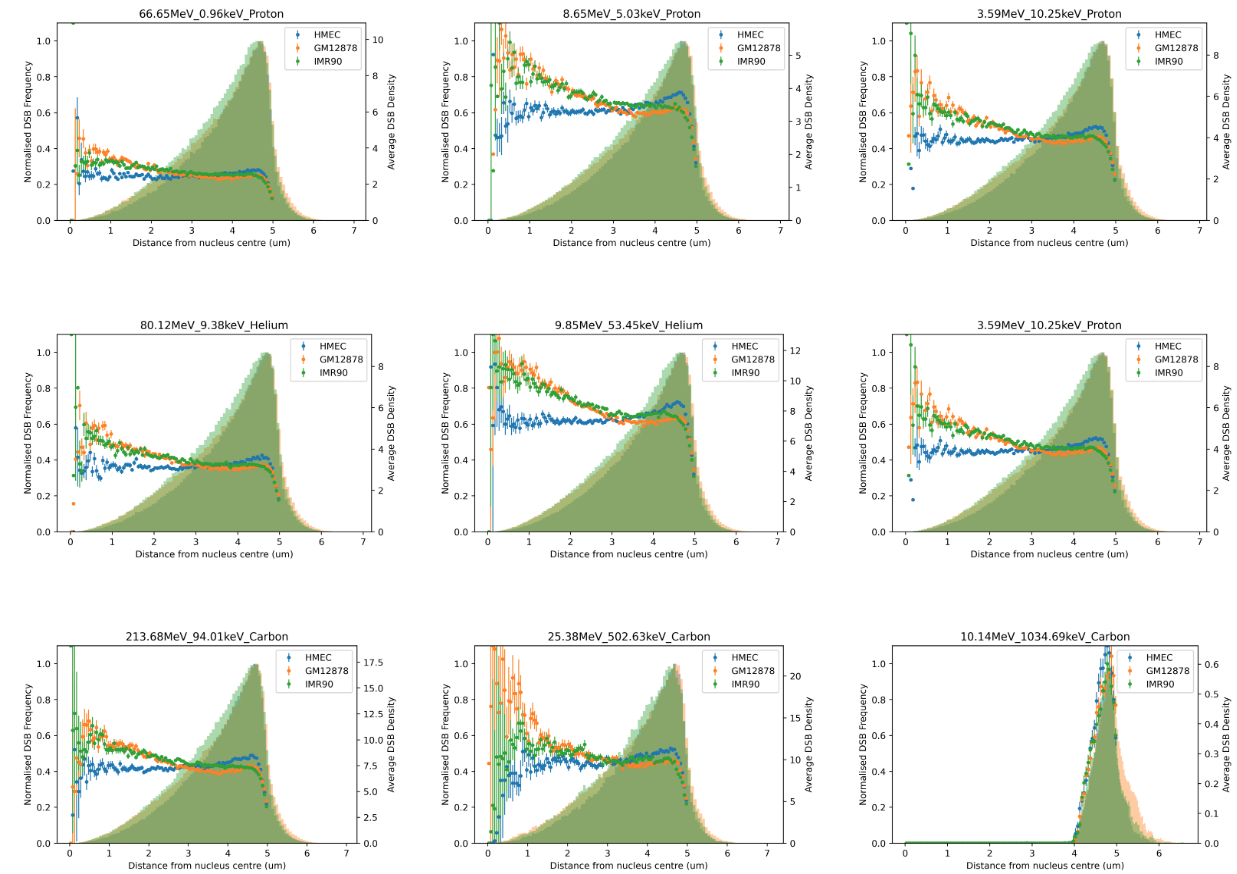

Supplement: S5 Fig — Dual axis plot—left y-axis shows the histogram plot of the Normalised DSB frequency and right y-axis is the corresponding average DSB density for the same x-axis bin per geometry. Both are given as a function of distance from the nucleus centre. The cell types are all solved for a spherical nucleus and do not include LADs. The DSB frequency was normalised to the maximum number of DSBs within any bin for a given cell type. DSB density is calculated as the average number of DSBs per geometry (N = 200) within a bin divided by the volume (μm3) of the spherical shell of the bin. Error bars in the DSB density are the standard error in the mean for all 200 geometries for each cell type. (TIF) [file pcbi.1008476.s005.tif]

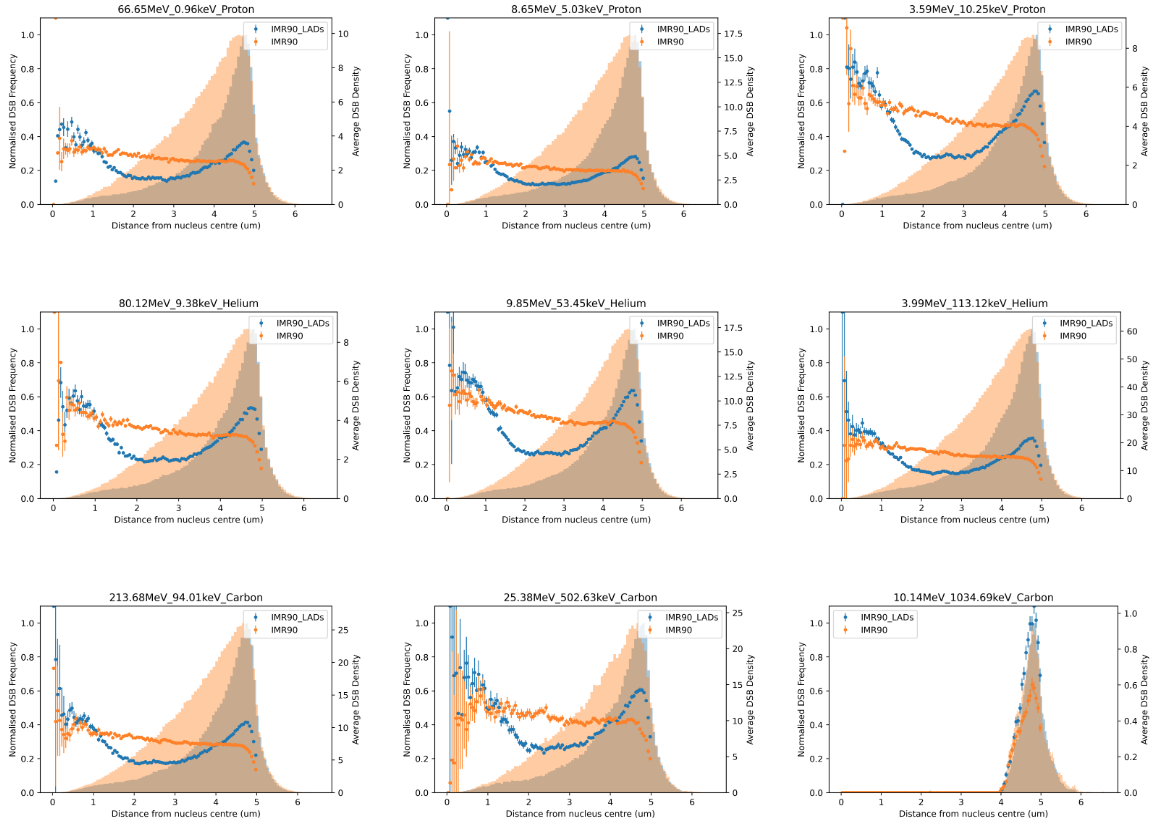

Supplement: S6 Fig — Dual axis plot—left y-axis shows the histogram plot of the Normalised DSB frequency and right y-axis is the corresponding average DSB density for the same x-axis bin per exposure. Both are given as a function of distance from the nucleus centre. Comparison between IMR90 with and without LADs constraints for a spherical nucleus. The DSB frequency was normalised to the maximum number of DSBs within any bin for a given cell variant. DSB density is calculated as the average number of DSBs per geometry (N = 200) within a bin divided by the volume (μm3) of the spherical shell of the bin. Error bars in the DSB density are the standard error in the mean for all 200 geometries for each cell variant. (TIF) [file pcbi.1008476.s006.tif]

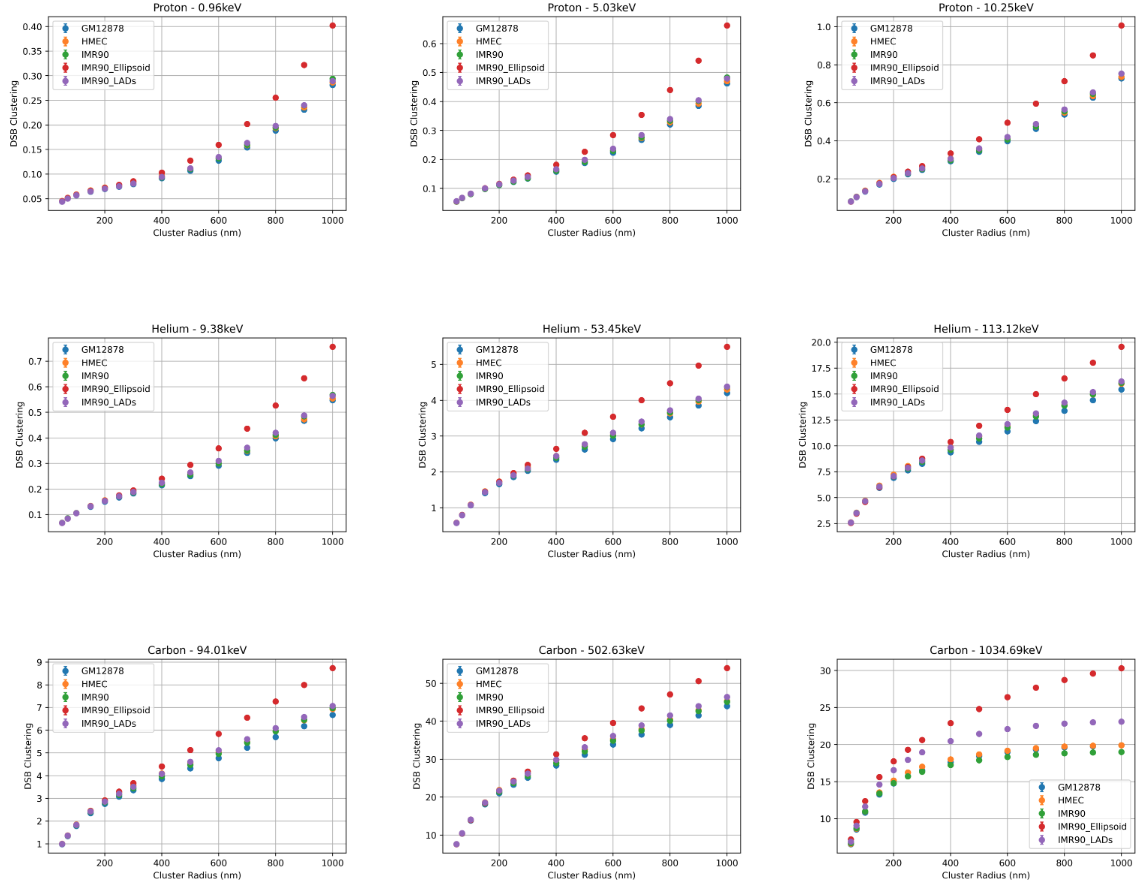

Supplement: S7 Fig — Double-strand break clustering as a function of the cluster radius for all cell-types and variants. Error bars are displayed as the standard error of the mean for 200 geometries for each cell-type and variant with each geometry having 50 independent exposures. (TIF) [file pcbi.1008476.s007.tif]

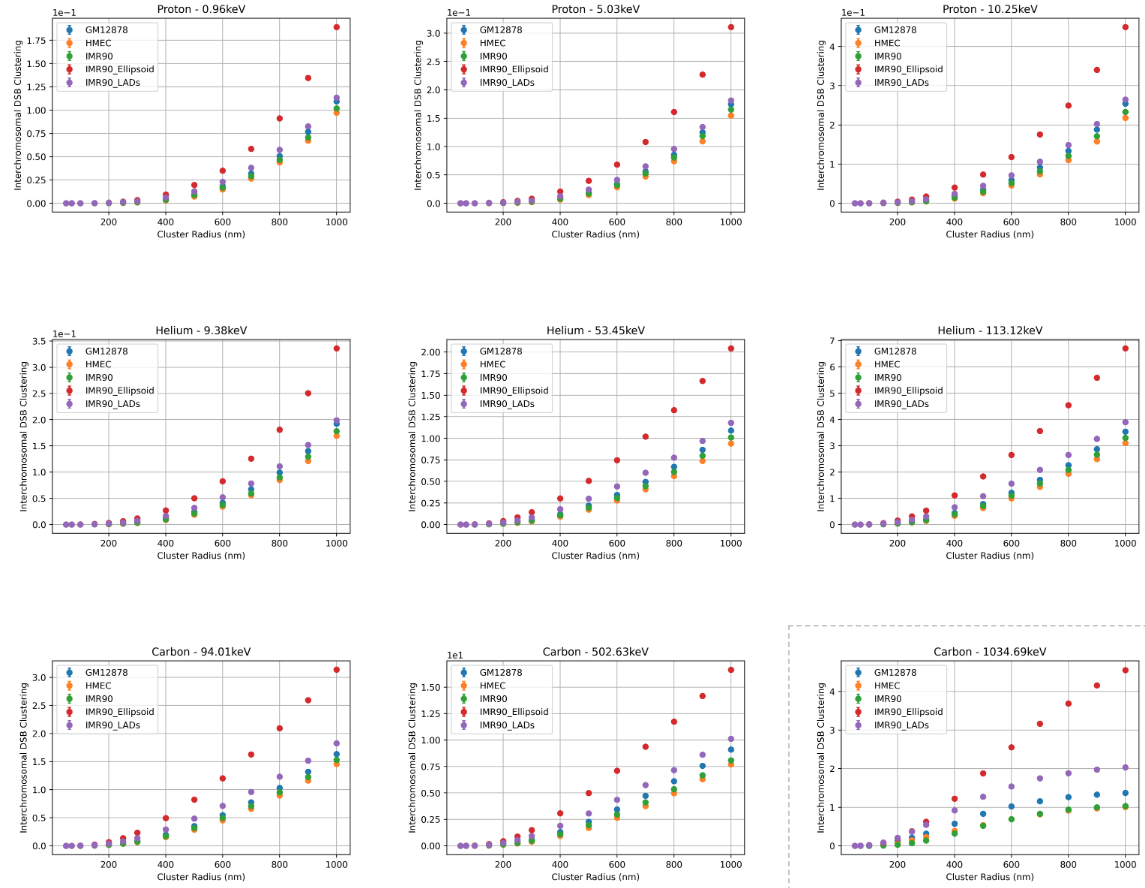

Supplement: S8 Fig — Double-strand break interchromosomal clustering as a function of the cluster radius for all cell-types and variants. Error bars are displayed as the standard error of the mean for 200 geometries for each cell-type and variant with each geometry having 50 independent exposures. (TIF) [file pcbi.1008476.s008.tif]

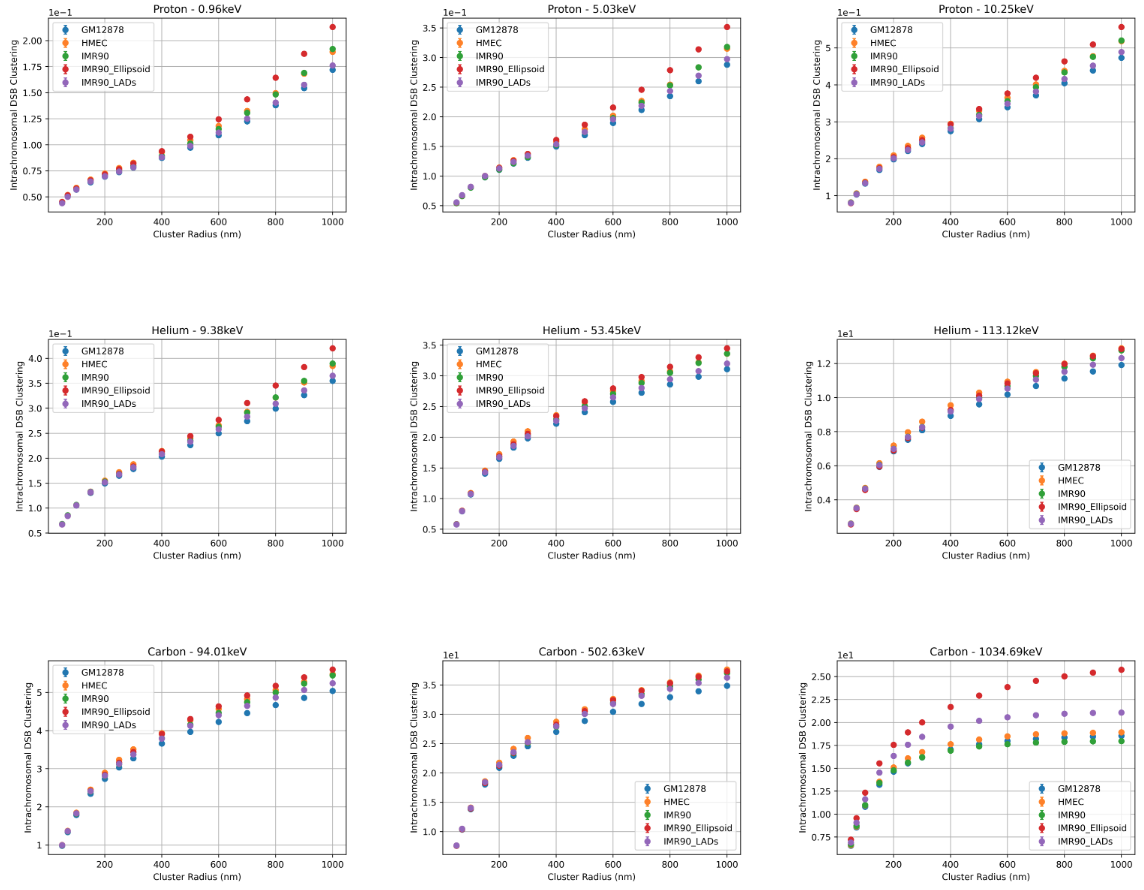

Supplement: S9 Fig — Double-strand break intrachromosomal clustering as a function of the cluster radius for all cell-types and variants. Error bars are displayed as the standard error of the mean for 200 geometries for each cell-type and variant with each geometry having 50 independent exposures. (TIF) [file pcbi.1008476.s009.tif]

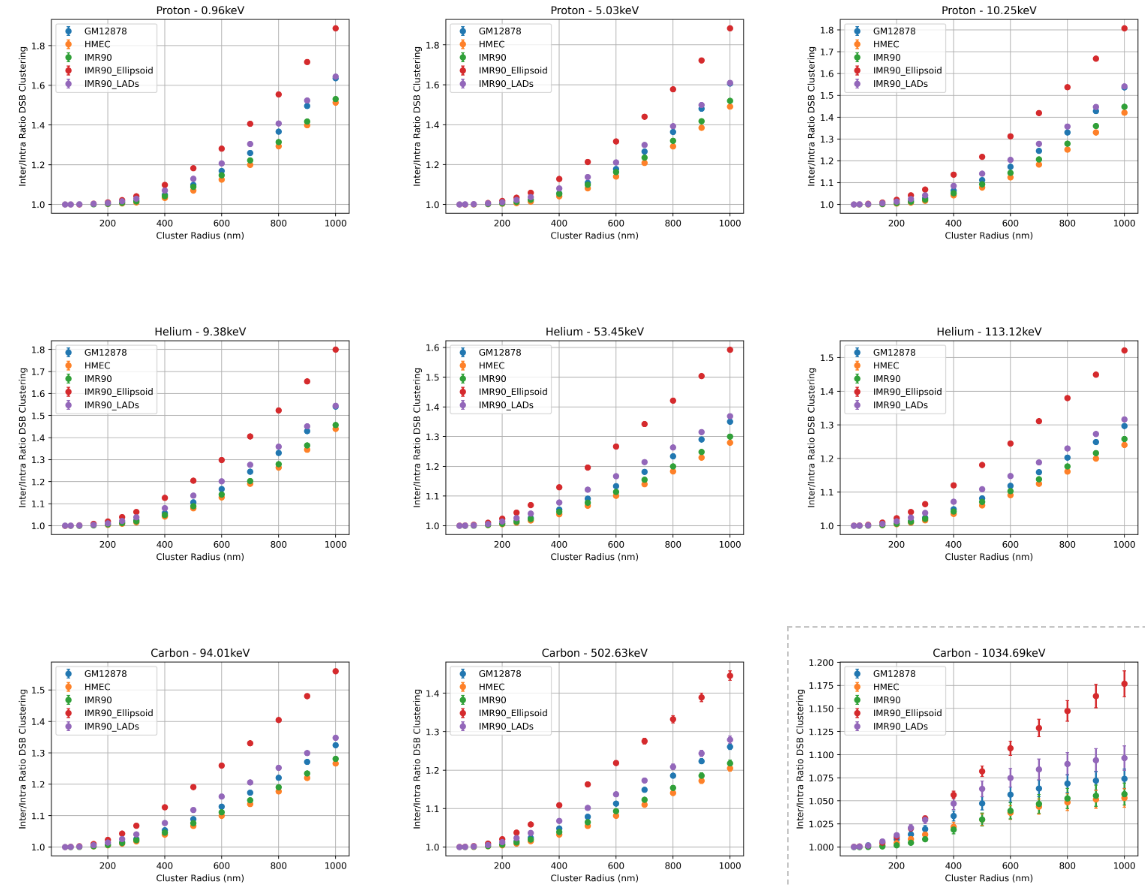

Supplement: S10 Fig — Double-strand break inter/intra chromosomal clustering as a function of the cluster radius for all cell-types and variants. (TIF) [file pcbi.1008476.s010.tif]

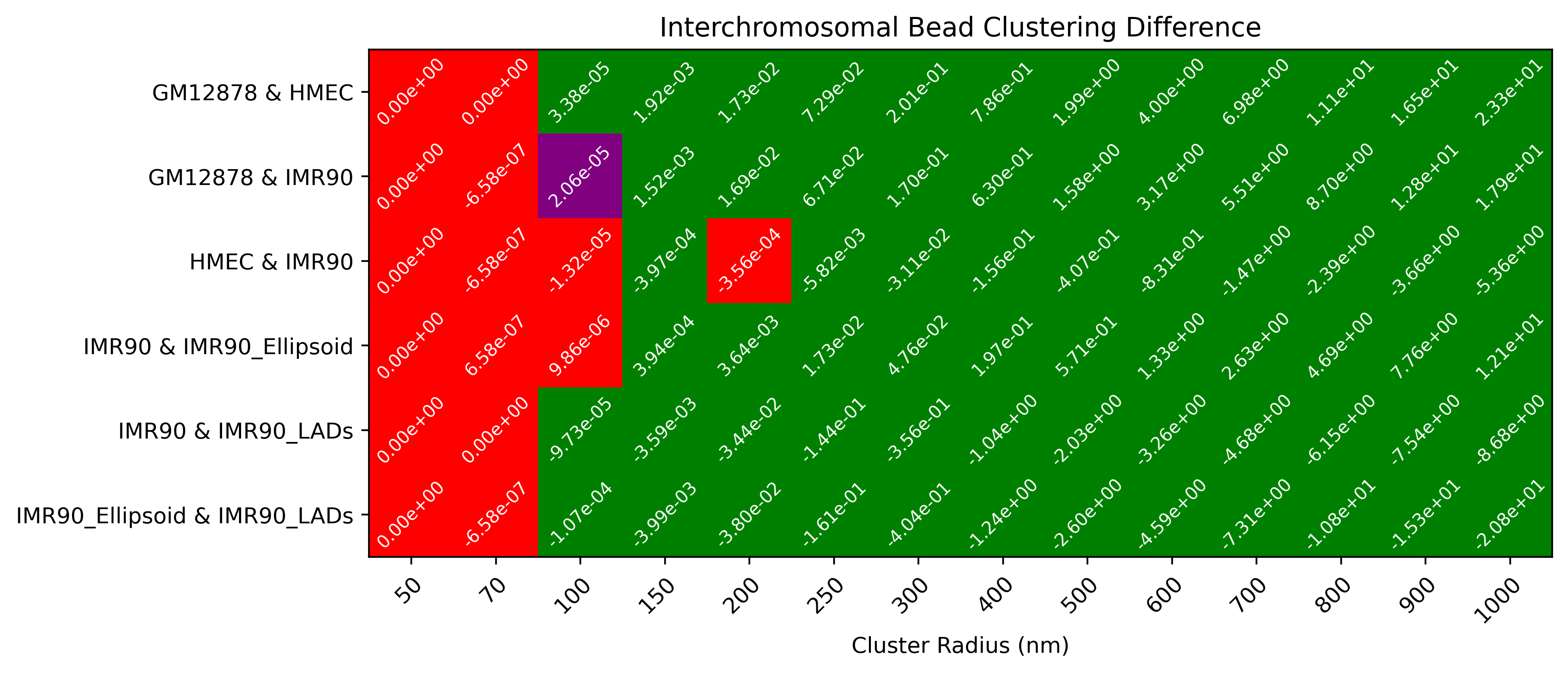

Supplement: S11 Fig — False discovery rate adjusted P-values from a 2-sided Kolmogorov-Smirnov test on the interchromosomal bead clustering values for the different cell-types and variants. Colour coding for adjusted P-values at varying thresholds: red (P > 0.05), purple (0.05 > P > 0.01), yellow (0.01 > P > 0.001) and green (P < 0.001). In this case, distributions with adjusted P-values < 0.05 will be considered as having significant statistical difference to one another. Each of the tested distributions had 200 geometries per cell-type or variant group. (TIF) [file pcbi.1008476.s011.tif]

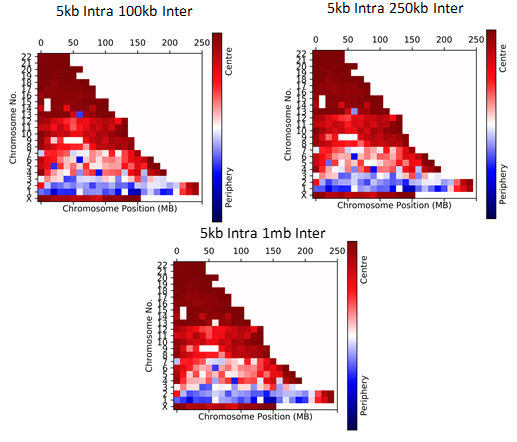

Supplement: S12 Fig — Bead positioning between periphery and central locations for a range of different interchromosomal contact resolutions at the finest available intrachromosomal contact resolution. Each category consists of 200 geometries created from the corresponding gtrack file created from using different analysis resolutions. (TIF) [file pcbi.1008476.s012.tif]

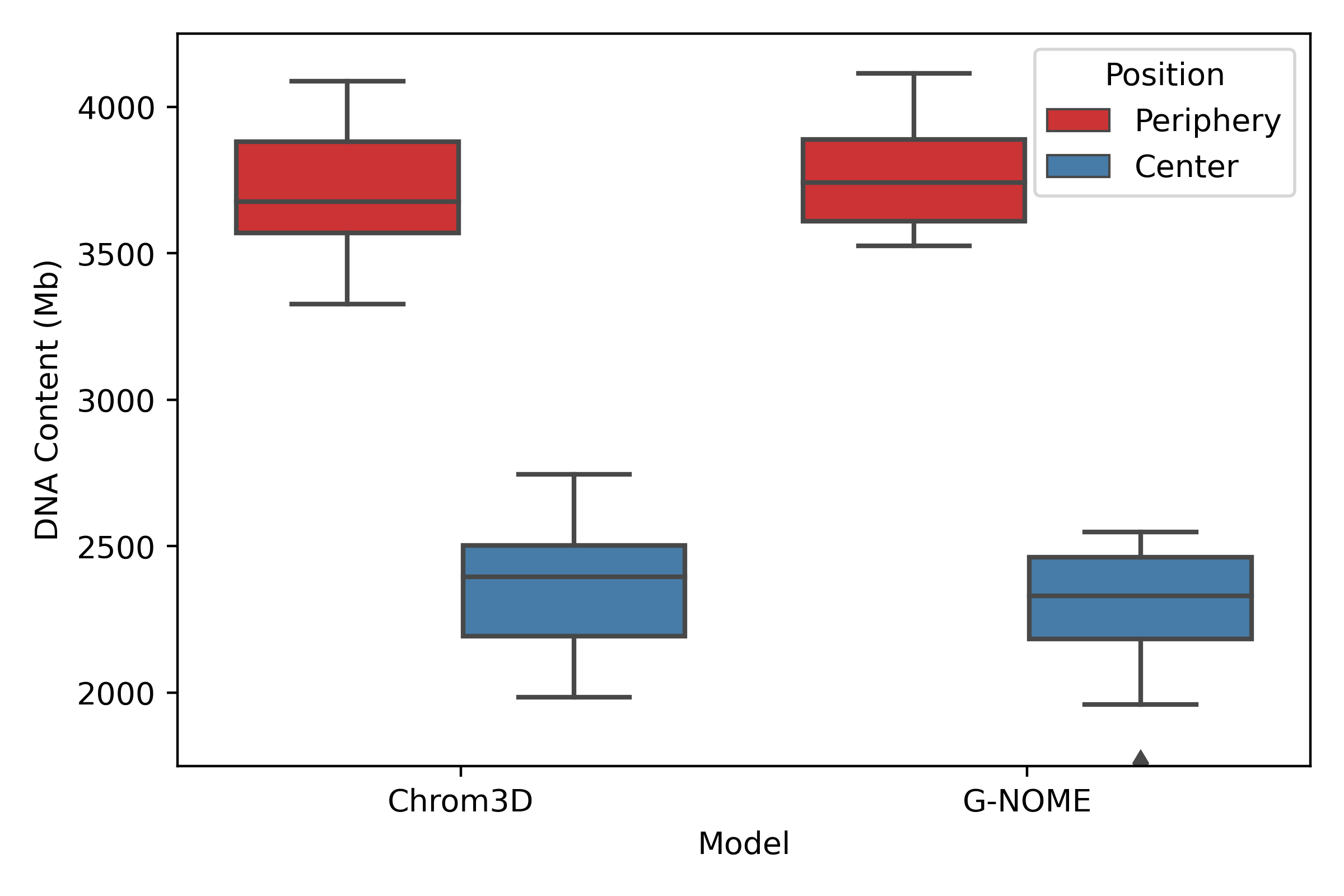

Supplement: S13 Fig — Box plots of the DNA content positioned either in the peripheral half or central half of the cell nucleus volume. These results are for 50 geometries from G-NOME and 50 geometries from Chrom3D (v1.0.2). In both models the same input IMR90 noLADs gtrack file was optimised for 1 million iterations, 5-micron nuclear radius and 0.15 occupancy volume. (TIF) [file pcbi.1008476.s013.tif]

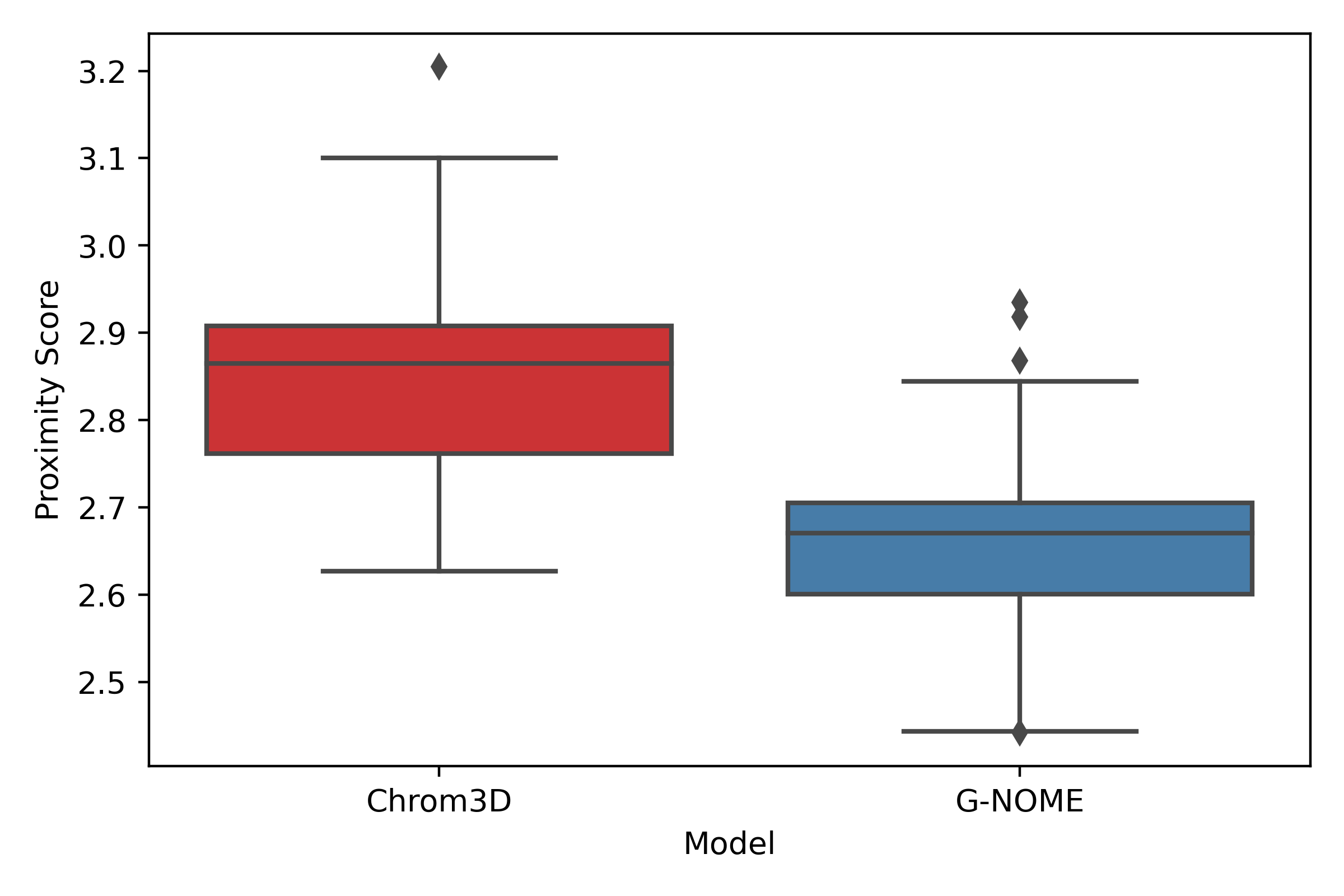

Supplement: S14 Fig — Box plots of the proximity scores which is the average Euclidean distance between TADs which have a constraint to be proximal other TADs (lower value indicates a better optimisation of the contact constraints). To put these differences into perspective for a randomly distributed geometry where the proximity score is approximately 12. These results are for 50 geometries from G-NOME and 50 geometries from Chrom3D (v1.0.2). In both models the same input IMR90 noLADs gtrack file was optimised for 1 million iterations, 5-micron nuclear radius and 0.15 occupancy volume. (TIF) [file pcbi.1008476.s014.tif]

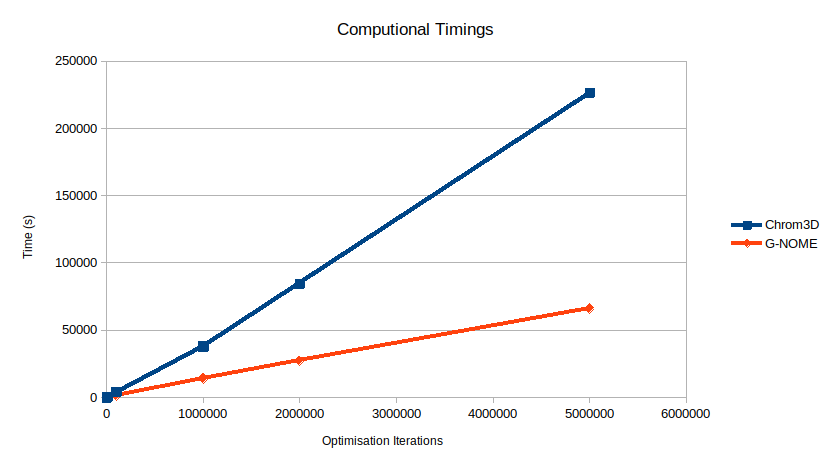

Supplement: S15 Fig — Timing performance for a nominal single IMR90 spherical cell 3D geometry generation using both G-NOME and Chrom3D (v1.0.2). In this case for 2 million iterations (the number used for the evaluation of different cell types) is 23.5 hours in Chrom3D and 7.6 hours in G-NOME. (TIF) [file pcbi.1008476.s015.tif]

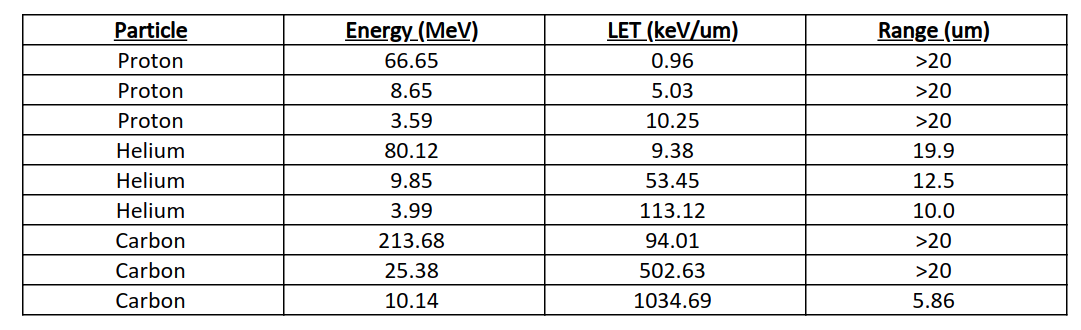

Supplement: S1 Table — Examination of particle range travelling through 20μm of water. Simulation was carried out for each energy with the furthest depth of each particle scored and averaged for 100 single-particle transversals. Particles which transverse beyond the 20μm of water are simply signified as having ranges beyond 20μm. (TIF) [file pcbi.1008476.s016.tif]
