# Supplementary material for: Hi-C implementation of genome structure for in silico models of radiation-induced DNA damage
Source: PLoS Comput Biol. 2020 Dec 16;16(12):e1008476. doi: 10.1371/journal.pcbi.1008476 (PMC7773326; doi:10.1371/journal.pcbi.1008476)
Supplement: Attachment — Submitted filename: AuthorResponse.pdf [file pcbi.1008476.s026.pdf]

Dear Reviewers,

Firstly, the authors would like to thank you for the detailed and constructive comments you have provided. We believe these comments have been instrumental to improve clarity and the scientific merit of the new revised manuscript. Below we have the individual responses for each of your comments. The colour coding for this response will be as such:

Reviewer comments.

Author responses.

Manuscript additions (including starting line).

Reviewer 1

1. Although the focus of the manuscript is not on the 3D genome modeling and its validation, the results heavily depend on the exact fact. The authors in the manuscript re-implemented the Chrom3D program as G-NOME to model the 3D genome organization from Hi-C data. They did mention some differences between the G-NOME and Chrom3D programs but without any comparative result. It is not clear to me about the novelty of re-implementing the original algorithm in some other programing language without any clear evidence of benefit. The modeling of ellipsoid geometry seems interesting as it is a novel feature highlighted by the authors to the G-NOME program, but the authors didn't provide any information on how they generated ellipsoid models and their biological validity as compared to spherical ones. Figure 1d shows the chromatin position of beads from IMR90-spherical, w/LADs, and IMR90-ellipsoid model. IMR90-ellipsoid, as compared to the spherical model shows very few beads near the nuclear periphery, is this only an optimization issue? I would suggest the authors correlate the model with LAD and gene-expression information to validate the ellipsoid model and the overall improvement of the G-NOME program. What are the "custom optimization" routines in G-NOME? And how they affect 3D modeling? Where are the generated 3D models?

G-NOME was developed as a re-implementation of the Chrom3D due to wanting to create something specifically for the track-structure modelling community in mind. Through the creation of a new software package we hope to cater more closely the needs of future radiobiology modelling which we believe are distinct to the original idea of Chrom3D. For a comparison we have added two additional supplementary figures which compares the bead arrangement between Chrom3D and G-NOME (Fig. S13-14) which have been referenced in the method section where we detail G-NOME. However, G-NOME does offer some advances to Chrom3D which include speed-up, ellipsoid nuclei and custom optimisation routines which have been detailed below:

The speed-up of G-NOME were made in the algorithm for checking for bead clashing. The differences in run time for a nominal example case are graphed below. This graph has been included in the supplementary material (Fig. S15) and is referenced to in the Methods section. This example is for the same gTrack file inputted into both models. In this case for 2 million iterations (the number used for the evaluation of different cell types) is 23.5 hours in Chrom3D and 7.6 hours in G-NOME.

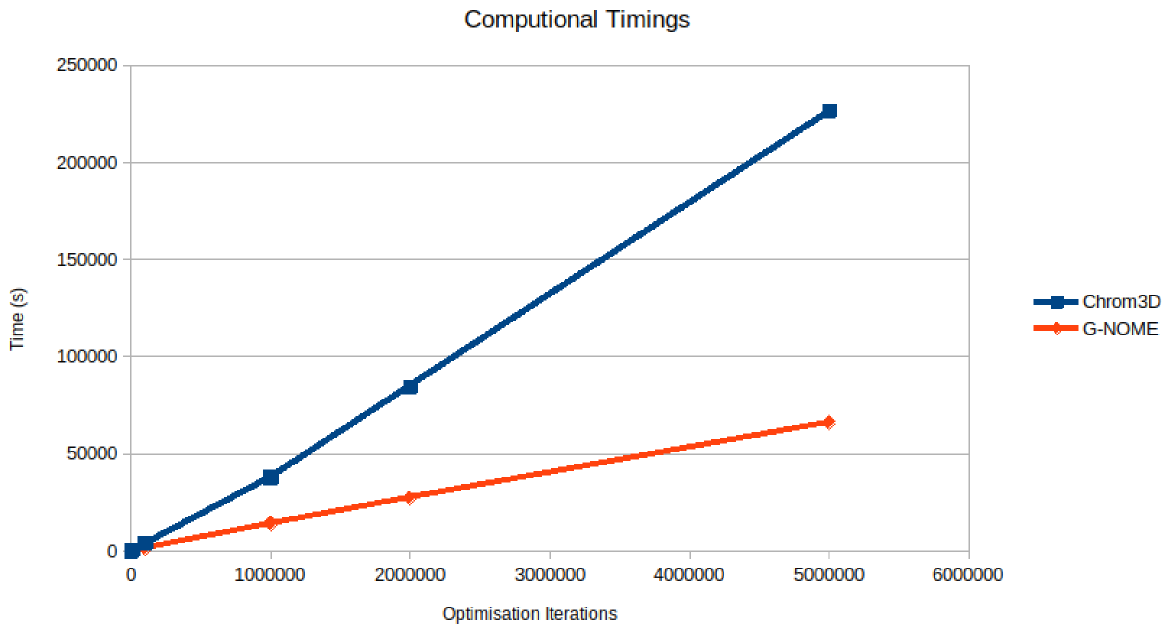

We agree with the observations made by the reviewer that in the Ellipsoid geometry solutions less beads are positioned in the periphery. To increase the clarity of this finding we have added the percentage of DNA content found in the periphery volume on the heatmaps of the chromatin positioning analysis (Fig 1d and Fig 2d). This is due to difficulties in optimising such a flattened cell, the periphery status is assigned to a bead if the central location of the bead is found in the periphery shell of the nuclear volume. As the nucleus becomes increasingly flattened it becomes increasingly hard to place a bead within this volume whilst not incurring a penalty from the nucleus constraint during optimisation (due to beads protruding from the nucleus due to their size). Therefore, we compare the fraction of beads outside the nucleus for all cell variants (Fig 1b) to check the optimization was fair for comparison between each of the cell variants. After your comment we have further evaluated this in our comparisons made in Fig 3, we have added a proximity score (Fig 3f) for each cell type and variant which measures the average Euclidean distance between beads which have constraints with one another to check that the Hi-C optimisation objectives achieved similar levels between groups. We have also added a boxplot (Fig 3g) for the amounts of DNA content either peripheral and centrally located for each cell type and variant. The details of these results have been discussed in the results section under the Comparison of Hi-C Genome Organisation and Pseudo-Random Organisation subsection.

The custom optimisation routines are available in G-NOME but have not been used within the results of the paper. This simply refers to the ability to change optimisation objectives during runtime of G-NOME. Due to Python being an interpreted language it is possible for the user to change constraints used by the optimiser at any stage of the optimisation process. For example, the user may want to optimise the beads meeting their Hi-C constraints before switching on the constraints for all beads to fit within the nucleus. We have added clarity around the custom optimisation routines in the manuscript text.

Line 465 - "Custom optimisation routines refers to the ability to be able to dynamically alter optimisation constraints during a run time session. We believe this gives us additional flexibility that may be required in future work with G-NOME and radiobiological modelling."

2. I am not clear about the "two conditions" and assignments of the DSB, SSB described in the DNA simulation section. What is the "spatial sampling of 14.1%" means as the first condition? And how come the authors arrived at this exact number? In the second condition, probabilities corresponding to energies ranging from 5 to 37.5 eV are assigned. Is this a standard range of energies during simulation, and where are the references supporting the procedure? The author's talks about energy values in KeV and MeV range, how are these high energies converted to probability space? The 3D geometries are solved from a low-resolution Hi-C matrix, but how these low-resolution beads are used to approximate double or single-strand DNA breaks is not clear to me. The authors mention that DSB induction is assumed to follow a Poisson distribution, and chosen randomly with probability weighted according to the chromosome and bead size. The chromosome size remains invariable across cell types, and only the bead size at TAD resolution should provide some cell line specific weightage to determine the probability for DSB induction. But the 3D modeling of a genome from Hi-C data provides the spatial position of a bead relative to the nuclear lamina (Figure 1d). From previous studies, it is known that this relative position (periphery to central) is highly variable across genomes and should provide the most cell line specific information. The function to weigh this spatial positioning of a bead relative to the nuclear periphery and energy source while calculating the probability to induce DSB is missing, and I think this should affect the overall analysis.

We have added a paragraph to the methods section to give some background to where the two conditions arise. These conditions have been extensively evaluated in both ours and other members for the track-structure community publications.

The energy-based probability of damage induction is taken from PARTRAC (Friedland et al 2003) and has been implemented in several other models. Previous modelling work tended to use an energy threshold of 17.5 eV, though several studies (most notably the experimental work of Sanche 2002) showed that strand breakage can be induced by very low energy electrons.

The 14.1% sensitive fraction was applied to energy depositions occurring within a Hi-C bead. The number was chosen to reproduce DSB yields seen in our previous work (Henthorn & Warmenhoven et al 2018), which in turn was fit against several other models (notably Meylan et al 2017). The Meylan model was fit against experimental data and compared to PARTRAC (indirect validation).

For clarity the Poisson distribution for DSB induction is only used to model Cobalt-60 (x-ray) irradiation. For all particle irradiation the track of the charged particle dictates the overall pattern of induced radiation damage and therefore track-structure simulation using Geant4-DNA was used.

In this study we only had lamina associated domain information for the IMR90 cell line. Therefore, we were unable to compare multiple cell lines with this additional lamina constraint for the proposed probability to induce DSB as a function of spatial positioning analysis. In the future we would like to evaluate this metric across multiple cell lines in conjunction with heterochromatin and euchromatin status.

“Simulation of DNA Damage after Proton Irradiation”; W Friedland et al; 2003

“Low-Energy Electron Damage to DNA and its Basic Constituents”; L Sanche; 2002

“In Silico Non-Homologous End Joining Following Ion Induced DNA Double Strand Breaks Predicts That Repair Fidelity Depends on Break Density”; N Henthorn & J Warmenhoven et al; 2018

“Simulation of early DNA damage after the irradiation of a fibroblast cell nucleus using Geant4-DNA”; S Meylan et al; 2017

Line 509 - “The spatial sampling of 14.1% corresponds to a sensitive fraction of the bead and was determined to reproduce DSB yields seen in our previous work (Henthorn2018). This fit is in good agreement with other models (Meylan2017, Friedland2003) which have been validated against experimental data. The energy-based probability of damage induction was adapted from PARTRAC (Friedland2003) and is based on studies of DNA strand breakage after exposure to very low energy electrons (Sanche2003).”

3. Figure 2c shows a higher cluster density for GM12878, while the other two don't differ too much. Is this because of the sequencing depth difference among the Hi-C experiments? Are the Hi-C data downsampled or normalized before 3D model building to reduce experimental biases? How does the DSB/SSB yield/pattern vary as a function of spatial positioning in the nucleus relative to the periphery?

All Hi-C data was taken from the widely utilised data from Rao et al 2014. This is the same sample data used by Paulsen et al 2017 for the Chrom3D publication and was processed in the manner described by the protocol paper of Paulsen et al 2018. The Hi-C data was taken at the base pair delaminated resolution of 50 kbp for the intra-chromosomal matrix and 1 Mbp for the inter-chromosomal matrix. The raw matrices are taken and processed as specified in the Chrom3D protocol. We examined if the 3D models changed with differing input contract matrices and found the Chrom3D protocol to produce gTrack files was insensitive to it for the range tested (5kb – 50kb intra-chromosomal and 100kb – 1Mbp inter-chromosomal). We have ensured that this explained within the methods section under “Preparation of Hi-C data”.

A 3D Map of the Human Genome at Kilobase Resolution Reveals Principles of Chromatin Looping; S Rao et al; 2014

Chrom3D: three-dimensional genome modeling from Hi-C and nuclear lamin-genome contacts; J Paulsen; 2017

Computational 3D genome modeling using Chrom3D; J Paulsen; 2018

We have included details on how the DSB yield changes as relative to the periphery in supplementary figures S5 and S6. We give the result for each particle energy and show the difference between the cell types (Fig. S5) and the change when using LADs (Fig. S6). We now mention these results in the results section under the Simulated DNA Damage Yields in Hi-C Geometries section.

Line 237 - “To analyse how the yields of damage changes relative to the position within the cell nucleus both the DSB normalised frequency and DSB density has been plotted for each cell type (Fig. S5), which show minimal change between the cell types. The effects of adding lamina-based constraints in the IMR90-LADs geometries were evaluated against their counterpart IMR90 geometries, which do not have such constraints, in the same manner (Fig. S6). The increased positioning of beads towards the periphery does cause a notable effect for the damage distribution, with higher levels of DSB density located at the periphery.”

4. The pseudo-random geometry provides a good control to shows the overall importance of 3D geometries generated from Hi-C data (Figure 3b), but this is missing from figure 4b-c where they show induction of DSBs, SSBs/Gy as a function of energy and also in Fig S4. A baseline is always required to understand the improvement. Figure 3 isn't referred within the main text. What is "backbones per DSB" and its importance described in Figure S1? Why isn't it dropping sharply as DSB/Gy? It isn't easy for a reader to understand a plot if the same axis definition varies across the paper. The cluster density in the manuscript can be defined as per number the beads or DSBs within a cluster radius. I would suggest the authors define the Y-axis clearly as either "bead cluster density" or "DSB cluster density"

at appropriate places. What is "DSB interchromosome cluster density", where is it defined? Looking just at the numbers from Fig S4, S5, and Fig 5, it is clear that intrachromosomal DSB cluster density plays a major role and has more weightage then interchromosomal DSB cluster density. But the paper lacks the separate analysis of the intrachromosomal DSB part and its difference among cell types. Figure S4 "Carbon 1034.69Kev" shows a completely flattened IMR90-ellipsoid model; why is that?

The pseudo-random geometry was not included in figure 4b-c as it was concluded from this section that the yields of damage are unaffected by the spatial arrangement of the DNA content. Therefore, if added the points would simply align with the others in figure 4b-c.

We thank the reviewer for pointing this out, Figure 3 was incorrectly referenced in the results subsection Comparison of Hi-C Genome Organisation and Pseudo-Random Organisation. This has been corrected and is now referenced.

Regarding backbones per DSB. DSBs are formed when two DNA backbones on opposite sides of the helix are damaged and within 10 base pairs of each other. It is thought that the forces exerted on the structure of the DNA will then be enough to overcome the hydrogen bonds between the bases, causing the two ends to separate. However, as the density of energy depositions from the radiation increases, so does the probability that additional backbones are damaged within close proximity to these two initial breaks. These additional damaged backbones will then be incorporated into the DSB as the DNA strand will separate at the weakest point with hydrogen bonds retaining the rest of the material. At sufficiently high LETs this will cause a reduction in the total number of DSBs as more and more of the backbone damages are combined into the same DSBs. To demonstrate that this is what is occurring for the high LET carbon we graph Figure S1 which shows the average number of backbones per DSB as a function of LET and the average number of DSBs as a function of the same. The graph shows the expected trends, with the lines not being an exact reverse of each other as the probabilities aren't linearly related to each other.

We completely agree with the terminology needing to be specified clearly. Therefore, before every usage of clustering (formerly cluster density) we now either refer to bead (geometry) or DSB (damage). We have also unified the suffix term to interchromosomal and intrachromosomal. There is no longer interchromosome and this has become interchromosomal. This fits with the definition of terms section.

Whilst intrachromosomal clustering is more prevalent there is an increased interest in interchromosomal events due to the catastrophic nature of mis-repair between two different chromosomes. Interchromsomal aberrations are highly toxic to the cell and can lead to mitotic catastrophe. Therefore, the focus was predominately on intrachromosomal clustering due to the radiobiological interest. However, there is still interest in intrachromosomal mis-repair which can have a wide array of biological implications. Therefore, we have added the 2D plots of how this varies as a function of cluster radius in the supplementary. To make the reason for our focus on these clearer we have also added information on DNA mis-repair and chromosomal aberration in the introduction.

Upon initial submission we believed the yield drop for Carbon 1034.69Kev/um was due to the low number of tracks required to give 1Gy to the cell nucleus mostly depositing its energy outside of the nuclear beads. However, we have investigated this further and have identified that the decrease in damage yields was due to a positioning offset between the radiation source and the entrance of the nucleus. We have re-simulated this for Carbon 1034.69Kev/um on the ellipsoid geometries, which was the only energy affected by this issue and have corrected the graphs. The yield results are now much

more in line with the overall trends seen at different particles and energies. We have also now included these datapoints within Fig. 6b as they no longer skew the overall results.

Reviewer 2

1. The target audience of the manuscript includes two broad and separate groups of readers. One of them is knowledgeable in radiation-induced DNA damage but has only basic knowledge in Hi-C data analysis. The other is familiar with Hi-C data, but has only rudimentary knowledge of radiation-induced DNA damage. The Introduction could be expanded a bit, so that the manuscript would be more accessible to the latter group. In particular, the concepts of DSBs and interchromosonal translocations should be introduced there.

We have now added this information to the introduction. We appreciate the observation that this was missing.

Line 236 - This is due to the radiobiological response being majorly driven by damage to the DNA structure. The formation of double-strand breaks (DSBs), which is where the sugar-phosphate backbone of the DNA is broken on both sides in close proximity (<10 bp) has been shown to correlate strongly with cellular survival (Dugle1976, Wang2018). This is thought to be partially caused by DSBs giving rise to the possibility of chromosomal interchanges, whereby chromosomes that misrepair can form a whole variety of chromosome aberrations (Savage1976). It is believed that a major factor for misrepair events is the mobility and spatial distribution of DNA break ends (Sachs2009, McMahon2016, Warmenhoven2020), increasing the importance of chromosomal arrangement on cell fate. One of the more lethal chromosome aberration types are those that involve two different chromosomes being misrepaired and plays a major role in the radiation-induced cell death (Ballarini2010, Sia2020). The probability for these types of chromosomal translocation to occur has been shown to relate to the intermingling of the different chromosomes (Branco2006). Therefore, there is an innate interest in being able to accurately predict the spatial distribution of interchromosomal DNA break ends as this will inform us to the probability of inducing interchromosomal aberrations.

2. A central term in the paper is "cluster density" (CD). It is locally defined as the number of objects that fall within a given radius, and then averaged for all objects in the simulation. This term is closely related (precisely speaking, proportional) to Ripley's K function, commonly used in testing spatial point patterns. (K function was introduced in Ripley, B. D. (1979). Tests of “Randomness” for Spatial Point Patterns. Journal of the Royal Statistical Society - Series B (Statistical Methodology), 41(3), 368–374; you can see https://wiki.landscapetoolbox.org/doku.php/spatial_analysis_methods:ripley_s_k_and_pair_correlation_function for a gentle introduction).

I found the term "cluster density" misleading, because it is not a density (it is not normalized by the volume). As far as I am aware, this term is newly introduced in this manuscript. The authors should consider one of the following:

(A) Define "cluster density" as a density (the number of objects that fall within a given radius, divided by the volume of the ball of this radius).

(B) Use Ripley's K function instead.

(C) Use a different word, say "count", instead of "density".

It would be interesting to see what Fig. 1c and 2c look like when plotting the density as defined in (A).

The term “cluster density” was introduced in our previous work (Henthorn & Warmenhoven et al., 2018). However, we agree with the reviewer that this term is innately confusing as it is not actually a density measure. Therefore, we have now altered this term to simply clustering. We would like to keep the term at its current definition so it can be related back to previous work. Furthermore, we believe this current definition is easily interpretable as it expresses the average number of DSB break-ends available for a given repair radius.

We were unfamiliar with Ripley’s K function and would like to thank you for bringing it to our attention. This is a good statistical method to evaluate the clustering or dispersion of the resultant G-NOME model. Therefore, we have included this analysis in the manuscript (Fig 3e) which provides a nice validation that the pseudo-random geometry is completely spatially random, and the other geometries have varying levels of clustering. We have also added details in the Statistical Analysis section of the Methods to detail our Ripley K function calculation.

Line 342 - “To spatially analyse the geometries the normalised Ripley-K function was calculated for the 3D geometries (Fig. 3e). The pseudo random geometry resulted in a straight y=0 line for all cluster radii, suggesting it is completely spatially random. The IMR90 Ellipsoid geometry displayed an increase in clustering with a positive non-linear trend at a magnitude that was distinct to the other geometries. Whereas, the remaining geometries showed a lesser, but still non-linear separation to the y=0 line of complete spatial randomness, suggesting some amounts of clustering, but less than the IMR90 ellipsoid geometry.”

Line 570 - “The Ripley-K function was calculated for the 3D distrubtion of polymer beads for each 3D geometry produced by G-NOME. The calculation methodology follows the 3D implementation carried out by Jafari-Mamaghani et al (Jafari-Mamaghani2010). The equation is detailed below:

$$K(CR) = V_{Nuc}\frac{\sum_{i=1}^{n}\sum_{i\neq j} I[D(i,j) \leq CR]}{V_s n^2} \qquad (1)$$

where V_Nuc is the volume of the nucleus, CR is the cluster radius, n is the number of DSBs in the nucleus, V_s the edge correction term is the fraction of overlapping volume of the CR volume and the V_Nuc and I is the indicator function which will be either 1 if the condition D(i,j) ≤ t is true or will be 0, D(i,j) is the euclidean distance from DSB i to DSB j.”

To address your interest of seeing these plots as actual density plots, we have shown plots of Fig 1c and 2c as a correct depiction of the density below.

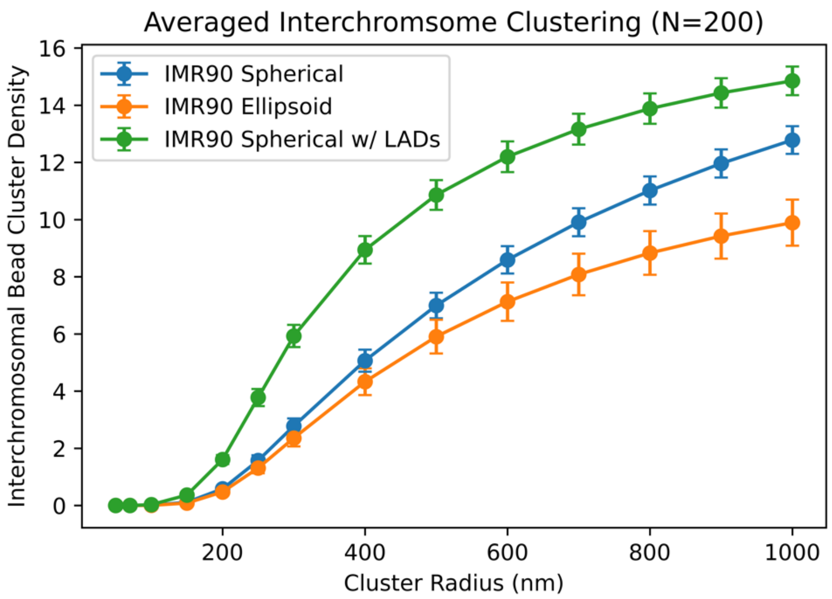

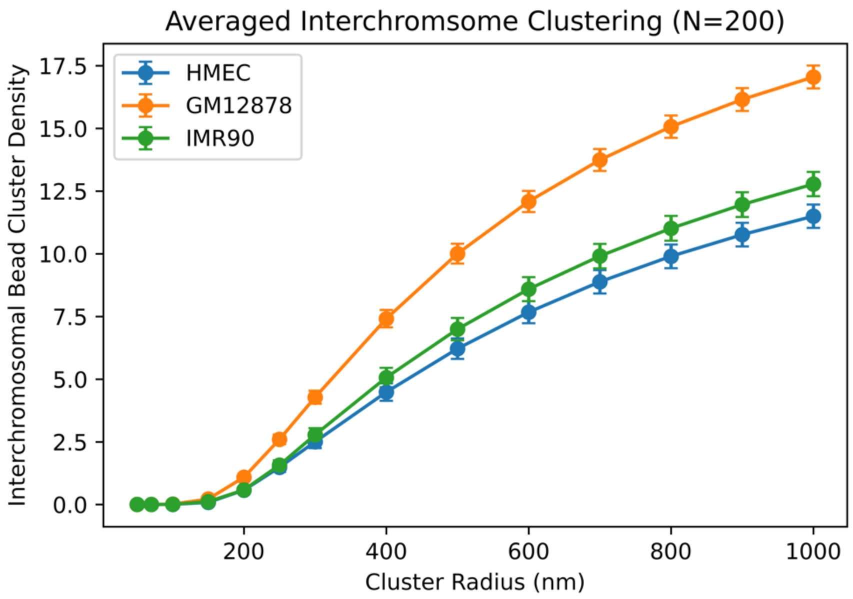

"In Silico Non-Homologous End Joining Following Ion Induced DNA Double Strand Breaks Predicts That Repair Fidelity Depends on Break Density"; N Henthorn & J Warmenhoven et al; 2018

3. Fig. 7 shows the p-values calculated in >600 statistical tests, without accounting for multiple hypothesis testing. This approach is not reliable, because it does not control for Type I error rate. I suggest to use false discovery rate (FDR) method for this purpose. More importantly, I would plot the effect size (e.g. the interchromosomal bead clustering values or ratio thereof) with a color or symbol depending on the (FDR-corrected) statistical significance. The same applies for Fig. S8. Also it is unclear what does the last sentence of Fig. 7 caption ("The value of...") refer to.

Fig 7 has been modified to match your recommendations. P-value colouring is now for the FDR adjusted p-value using the Benjamini-Hochberg correction. This has been reflected in the caption and the p-value adjustment has been added to the method's Statistical information section. The value displayed is not the difference between cell 1's and cell 2's interchromosomal clustering value. The

difference was used over the ratio as interchromosomal clustering values can be 0. The last sentence of Fig. 7 caption refers to each interchromosomal clustering value being the averaged value of 50 simulations of 1Gy exposures.

4. Radiation-induced DNA damage at the loci of interactions between two homologous chromosomes can also give rise to mis-repair and abnormal karyotype. The manuscript only considered interchromosomal clustering, but the description of raw data allows for including homolog interactions in the cluster density counting. This possibility (and the resulting observations) should be discussed in the manuscript.

We do count homolog interactions to the interchromosomal clustering as they do not belong to the same physical chromosome and can result in multiple centromeres if mis-repaired. We have updated this in the definition of terms section to make this clearer.

Minor Comments:

1. Acronyms such as DSB, SSB and LET should be spelled out at first use.

This has been added to the manuscript.

2. Fig. 1 and Fig. 2 show the same type of analysis, but comparing different underlying data (cell types and cell variants). It would be easier to comprehend the Results section if all the technical aspects were discussed along with explaining Fig. 1, so that the text discussing Fig. 2 could focus on the actual results.

We have attempted to move some of the technical parts towards Fig. 1, rather than repeating it for both Figures. However, we have done this cautiously as we want the information to understand Fig 2 to be readily available for the readers.

3. It is challenging to compare Fig. 1c and Fig. 2c to Fig. 3bc despite them presenting the same thing; log scale should be used consistently.

Fig. 3b now shows the same linear-linear plot as per Fig. 1c and Fig 2c.

4. It is nice to have an interactive 3D version of Fig. 5, but in the paper it would be helpful to show some 2D cuts (for a fixed value of LET and for a fixed value of cluster radius). This would allow the reader to get the main message without opening the 3D plots.

The 2D plots of Fig 5 have been added into the supplementary material.

5. Fig. 6b is very difficult to read, due to multiple shapes of symbols being hard to distinguish when they cluster together. I propose to use facets here, and plot different cell types/variants separately.

We have added black lines to represent the standard deviation of each metric with the intersection corresponding to the mean value for Fig. 6b. This should distinguish the cell types/variants. We would prefer not to put them on separate facets to allow for easy comparison with one another. The ability to read off exact values from the graph is not necessary, the figure instead highlights the overall grouping between cell types/variant.

6. It is implied that the beads in the polymer model have different sizes (as seen in Fig. 1 and 2a). The authors should add one or two sentences explaining how the model accounted for that (are the distances between the beads constant?).

We have added a line to the methods section to make this clear. The beads are connected in a chain so the distance between the edge of neighbouring beads are the same.

7. The MCMC model tries several bead movement types at random. It would be clearer to enumerate these movements types in the Methods.

Movement types have been added to the methods.

Line 509 - “The movement types are as follows:

1. Crankshaft - rotates a random number of beads around two fixed beads points.
2. Arm Rotation - from a random position along the chromosome rotate all beads to the end or start of the the chromosome. The start or end is chosen by random.
3. Arm Wiggle - from a random position along the chromosome re-position (via a self avoiding walk) beads to the end or start of the chromosome. The start or end is chosen by random.
4. Translation - move the whole chromosome by a random x, y, z between 0 and 1um.
5. Rotation - rotate the whole chromosome by a random amount.”

8. Lines 144-159: Fig. 3 should be referred to, instead of Fig. 2.

This has been corrected.
