# Supplementary material for: Hi-C implementation of genome structure for in silico models of radiation-induced DNA damage
Source: PLoS Comput Biol. 2020 Dec 16;16(12):e1008476. doi: 10.1371/journal.pcbi.1008476 (PMC7773326; doi:10.1371/journal.pcbi.1008476)
Supplement: Attachment — Submitted filename: AuthorResponse.pdf [file pcbi.1008476.s027.pdf]

Dear Reviewers,

Firstly, the authors would like to thank you for examining our responses and your diligence in re-reviewing the manuscript. We hope the below responses resolve any outstanding comments.

Reviewer comments.

Author responses.

Additions to the manuscript.

Reviewer 1

1. Figure S5 and S6 show the spatial distribution of DSB yields in different cell types. How the authors calculated the Normalized DSB frequency? Looking at the Normalized DSB figure, it appears that there is no effect of spatial positioning on DSB yield, but the density result speaks otherwise. Also, how the normalized frequency can have a value above 1 (10.14MeV_1034.69KeV_Carbon)?

Thank you for your keen observation. In the previous version, we had plotted the histogram as a density rather than the count, meaning the count was the area of the bar rather than the height of the bar. We have now amended this so that the height is now the normalised frequency (max being 1). Each bin has been divided by the bin of the highest number of DSBs for a given cell type. We have also updated the DSB density to be the Average DSB density per cell geometry (N=200) for each cell type. This is instead of the total DSB density, which was averaged per exposure, we believe this is more intuitive. We have added error bars (standard error in the mean N=200) to the DSB density which highlights the difficulty to saying that there is any change in DSB density between cell-types as a function of distance from the nucleus centre. We do however see a much more noticeable difference in DSB density between the IMR90 spherical and IMR90 LADs geometries, which we discuss in the manuscript. We do see an increase in the DSB frequency (bars) as a function distance from the nucleus centre, but this is due to each bin representing an increased volume, hence the need to contrast this with the relatively flat DSB density (markers). Below are the new plots and captions which have been amended in the supplementary:

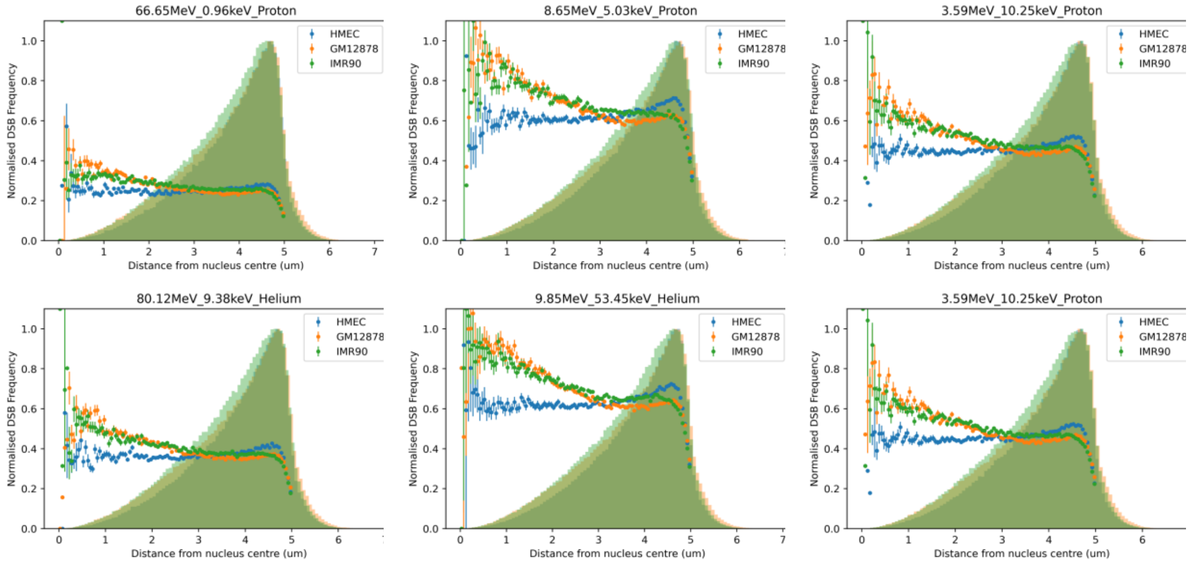

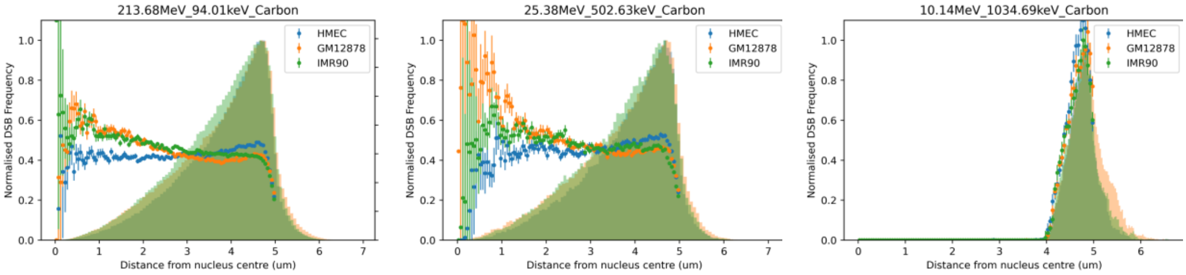

Figure S5 – Spatial distribution of DNA DSB yields for different cell types. Dual axis plot – left y-axis shows the histogram plot of the Normalised DSB frequency and right y-axis is the corresponding average DSB density for the same x-axis bin per geometry. Both are given as a function of distance from the nucleus centre. The cell types are all solved for a spherical nucleus and do not include LADs. The DSB frequency was normalised to the maximum number of DSBs within any bin for a given cell type. DSB density is calculated as the average number of DSBs per geometry (N=200) within a bin divided by the volume (um^3) of the spherical shell of the bin. Error bars in the DSB density are the standard error in the mean for all 200 geometries for each cell type. Title shows energy_LET_particle for each subplot.

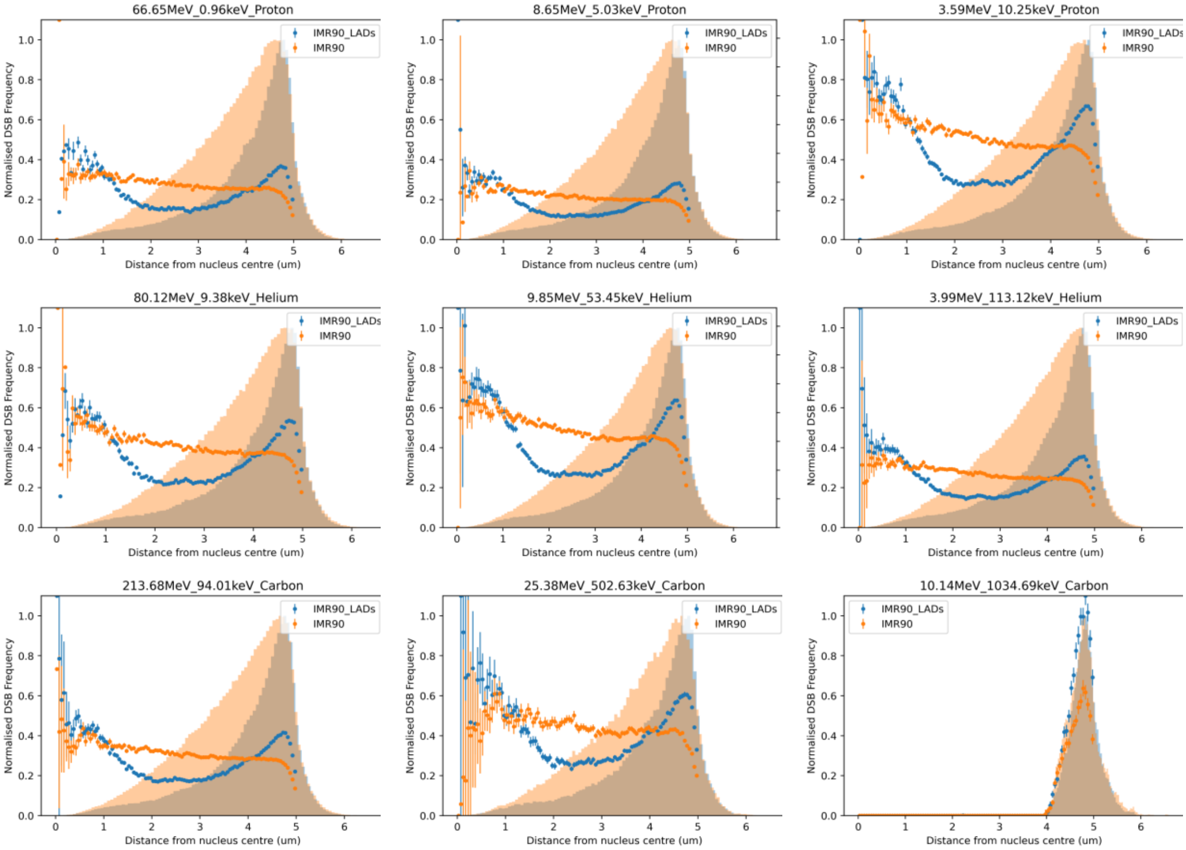

Figure S6 – Spatial distribution of DNA DSB yields for the addition of LADs. Dual axis plot – left y-axis shows the histogram plot of the Normalised DSB frequency and right y-axis is the corresponding average DSB density for the same x-axis bin per exposure. Both are given as a function of distance from the nucleus centre. Comparison between IMR90 with and without LADs constraints for a

spherical nucleus. The DSB frequency was normalised to the maximum number of DSBs within any bin for a given cell variant. DSB density is calculated as the average number of DSBs per geometry (N=200) within a bin divided by the volume (um^3) of the spherical shell of the bin. Error bars in the DSB density are the standard error in the mean for all 200 geometries for each cell variant. Title shows energy_LET_particle for each subplot.

2. Figure S13 and S14 shows the model quality generated by G-NOME and Chrom3D programs. Both figures show a significant difference in 3D models with the same set-up. Figure S13 shows a complete reversal of the total amount of DNA content placed in the Periphery and Center. Figure S14 shows a lower proximity score for Chrom3D models. It raises a serious concern about the quality of the models.

We have re-visited both sets of source code to try and evaluate what may have caused these differences and have identified a slight mistake in the set-up of our comparison case we used to make Figs. S13 & S14. This was due to a misunderstanding in Chrom3D's command-line option (--nucleus), which we initially thought added constraints for the beads to be within the nucleus was, pushing beads to the nuclear periphery. It appears that this is a redundant method in the Chrom3D source code as this is the same as the Chrom3D model constrains lamina-associated domains and the methods for both constraints are the same in the source code. Therefore, we have re-run the simulations without the nuclear constraints for both the G-NOME and Chrom3D models, using both the Hi-C TAD contact constraints in both models for the comparisons in Fig S13 & S14. As you can now see we get much better agreement between the two models and this is much more in line with what we expected. Whilst, the study does use nuclear constraints in the G-NOME model, we don't believe this should hinder the aim of the study, which is to compare between cell types and variants all produced using the G-NOME models using the nuclear constraints. We have added some detail in the methodology to explain this additional constraint in the G-NOME model. Please see below the new Fig S13 & S14.

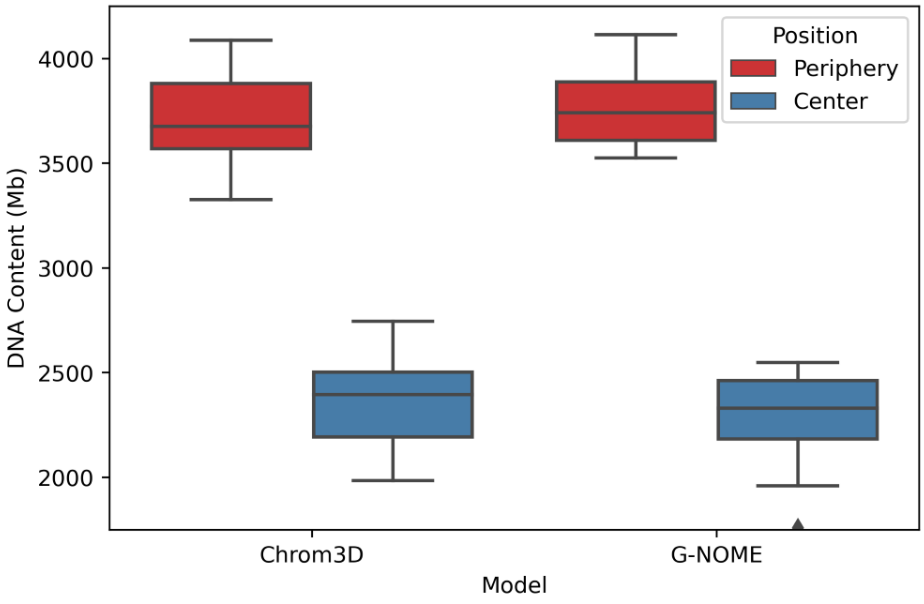

Figure S13 – DNA content position model comparison. Box plots of the DNA content positioned either in the peripheral half or central half of the cell nucleus volume. These results are for 50 geometries from G-NOME and 50 geometries from Chrom3D (v1.0.2). In both models the same input

IMR90 noLADs gtrack file was optimised for 1 million iterations, 5-micron nuclear radius and 0.15 occupancy volume.

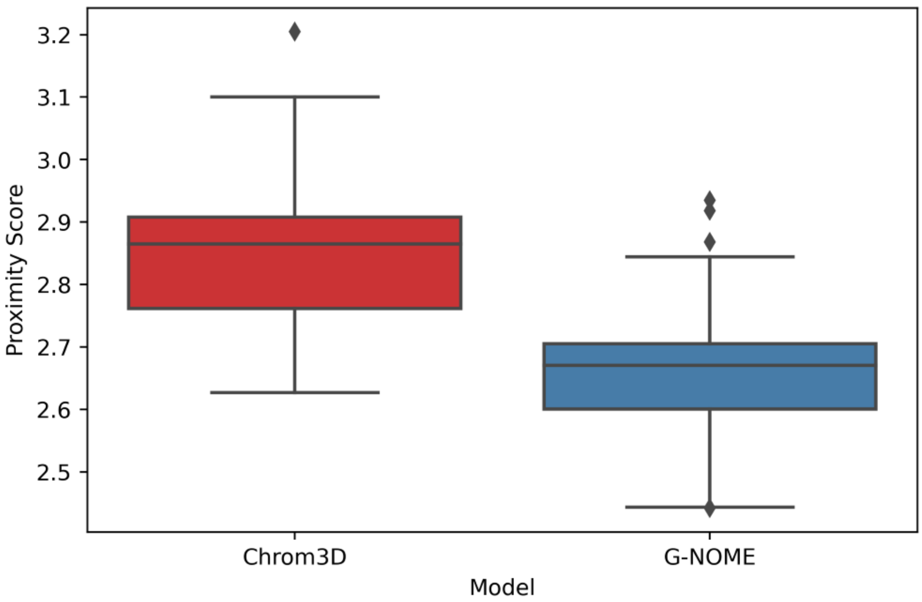

Figure S14 – Proximity score model comparison. Box plots of the proximity scores which is the average Euclidean distance between TADs which have a constraint to be proximal other TADs (lower value indicates a better optimisation of the contact constraints). To put these differences into perspective for a randomly distributed geometry where the proximity score is approximately 12. These results are for 50 geometries from G-NOME and 50 geometries from Chrom3D (v1.0.2). In both models the same input IMR90 noLADs gtrack file was optimised for 1 million iterations, 5-micron nuclear radius and 0.15 occupancy volume.

Also, for scale, we have plotted figure S14 on the same y-value maximum as the cell type and variant comparison in Fig3. With the pseudo random geometry having a value of approximately 12. This helps put any slight differences above into perspective. This scaled figure has not been included in the manuscript and is for demonstration purposes.

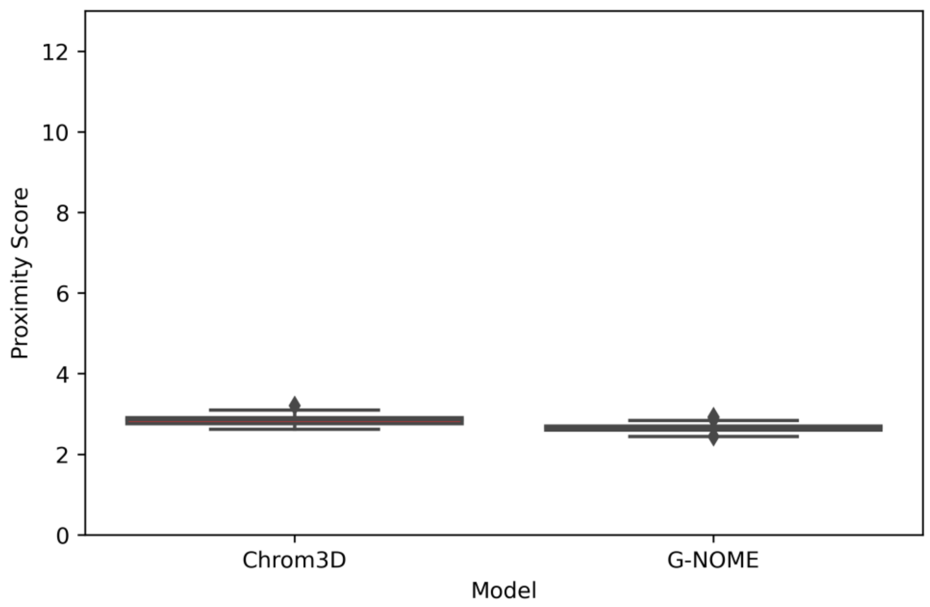

Below is the extract added to the methodology:

All geometries were solved using additional nuclear boundary constraints, which adds a cost based on if the beads were confined to the user-defined nuclear boundary. The costs applied to these constraints are 0 if the bead is within the cell nucleus and only occur cost on positioning outside of the nucleus based on the euclidean distance from the nuclear boundary. These constraints can be toggled when using the simulation run script provided through the flag "--ConstrainNucleus". These additional constraints were required as we wanted to preserve total volume across all cell geometries for the subsequent Geant4 simulation.

Reviewer 2

My only remaining comment is a minor detail: line colors in Fig. 3e should be the same as in Fig. 3b-d.

Thank you for highlighting this. To maintain consistency, we have updated Fig. 3e to match the line colouring of Figs. 3b-d. The new Fig 3 can be seen below:

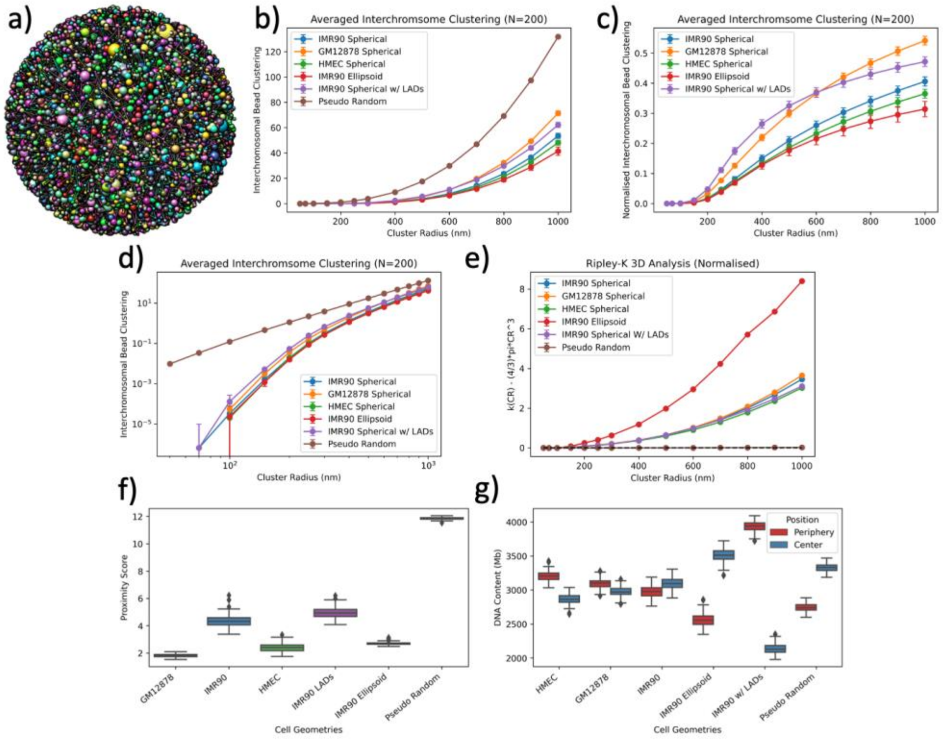
a)
b)
Averaged Interchromsome Clustering (N=200)
IMR90 Spherical
GM12878 Spherical
HMEC Spherical
IMR90 Ellipsoid
IMR90 Spherical w/ LADs
Pseudo Random
Interchromosomal Bead Clustering
Cluster Radius (nm)
c)
Averaged Interchromsome Clustering (N=200)
Normalised Interchromosomal Bead Clustering
Cluster Radius (nm)
d)
Averaged Interchromsome Clustering (N=200)
Interchromosomal Bead Clustering
Cluster Radius (nm)
e)
Ripley-K 3D Analysis (Normalised)
IMR90 Spherical W/ LADs
k(CR) - (4/3)*pi*CR^3
Cluster Radius (nm)
f)
Proximity Score
GM12878
IMR90
HMEC
IMR90 LADs
IMR90 Ellipsoid
Pseudo Random
Cell Geometries
g)
Position
Periphery
Center
DNA Content (Mb)
HMEC
GM12878
IMR90
IMR90 Ellipsoid
IMR90 w/ LADs
Pseudo Random
Cell Geometries
