# Supplementary material for: Hi-C implementation of genome structure for in silico models of radiation-induced DNA damage
Source: PLoS Comput Biol. 2020 Dec 16;16(12):e1008476. doi: 10.1371/journal.pcbi.1008476 (PMC7773326; doi:10.1371/journal.pcbi.1008476)
Supplement: Attachment — Submitted filename: Comments.docx [file pcbi.1008476.s025.docx]

Ingram et al., in this manuscript, integrate genome organization information from Hi-C data and radiation track structure modeling to understand DNA damage within cell-line specific genomes. Understanding the radiation-induced DNA damage has a critical relevance in different fields of research, especially cancer therapy or radiotherapy. The correct radiation dosage is extremely important to minimize the side effects of radiotherapy on healthy tissues while maximizing the effects on cancerous ones. Although a general safe amount of dosage is known, much improvement and understanding are still required in terms of the way the DNA is damaged to overcome the worst side-effects. The authors hypothesize that genome organizational features partially drive radiation effects on different cell types. Ingram et al., here coupled Hi-C inferred 3D geometries from three different cell lines (IMR90, HMEC, and GM12878) with radiation track modeling to understand the cell line specific radiosensitivity and the simulation results do show some pattern of DNA damage when exposed to radiation. I think the research work performed in this manuscript is important and adds clinical relevance to the utilization of Hi-C data. However, after going through the body of work I have the following major concerns -

1. Although the focus of the manuscript is not on the 3D genome modeling and its validation, the results heavily depend on the exact fact. The authors in the manuscript re-implemented the Chrom3D program as G-NOME to model the 3D genome organization from Hi-C data. They did mention some differences between the G-NOME and Chrom3D programs but without any comparative result. It is not clear to me about the novelty of re-implementing the original algorithm in some other programing language without any clear evidence of benefit. The modeling of ellipsoid geometry seems interesting as it is a novel feature highlighted by the authors to the G-NOME program, but the authors didn't provide any information on how they generated ellipsoid models and their biological validity as compared to spherical ones. Figure 1d shows the chromatin position of beads from IMR90-spherical, w/LADs, and IMR90-ellipsoid model. IMR90-ellipsoid, as compared to the spherical model shows very few beads near the nuclear periphery, is this only an optimization issue? I would suggest the authors correlate the model with LAD and gene-expression information to validate the ellipsoid model and the overall improvement of the G-NOME program. What are the "custom optimization" routines in G-NOME? And how they affect 3D modeling? Where are the generated 3D models?
2. I am not clear about the "two conditions" and assignments of the DSB, SSB described in the DNA simulation section. What is the "spatial sampling of 14.1%" means as the first condition? And how come the authors arrived at this exact number? In the second condition, probabilities corresponding to energies ranging from 5 to 37.5 eV are assigned. Is this a standard range of energies during simulation, and where are the references supporting the procedure? The author's talks about energy values in KeV and MeV range, how are these high energies converted to probability space? The 3D geometries are solved from a low-resolution Hi-C matrix, but how these low-resolution beads are used to approximate double or single-strand DNA breaks is not clear to me. The authors mention that DSB induction is assumed to follow a Poisson distribution, and chosen randomly with probability weighted according to the chromosome and bead size. The chromosome size remains invariable across cell types, and only the bead size at TAD resolution should provide some cell line specific weightage to determine the probability for DSB induction. But the 3D modeling of a genome from Hi-C data provides the spatial position of a bead relative to the nuclear lamina (Figure 1d). From previous studies, it is known that this relative position (periphery to central) is highly variable across genomes and should provide the most cell line specific information. The function to weigh this spatial positioning of a bead relative to the nuclear periphery and energy source while calculating the probability to induce DSB is missing, and I think this should affect the overall analysis.
3. Figure 2c shows a higher cluster density for GM12878, while the other two don't differ too much. Is this because of the sequencing depth difference among the Hi-C experiments? Are the Hi-C data downsampled or normalized before 3D model building to reduce experimental biases? How does the DSB/SSB yield/pattern vary as a function of spatial positioning in the nucleus relative to the periphery?
4. The pseudo-random geometry provides a good control to shows the overall importance of 3D geometries generated from Hi-C data (Figure 3b), but this is missing from figure 4b-c where they show induction of DSBs, SSBs/Gy as a function of energy and also in Fig S4. A baseline is always required to understand the improvement. Figure 3 isn't referred within the main text. What is "backbones per DSB" and its importance described in Figure S1? Why isn't it dropping sharply as DSB/Gy? It isn't easy for a reader to understand a plot if the same axis definition varies across the paper. The cluster density in the manuscript can be defined as per number the beads or DSBs within a cluster radius. I would suggest the authors define the Y-axis clearly as either "bead cluster density" or "DSB cluster density" at appropriate places. What is "DSB interchromosome cluster density", where is it defined? Looking just at the numbers from Fig S4, S5, and Fig 5, it is clear that intrachromosomal DSB cluster density plays a major role and has more weightage then interchromosomal DSB cluster density. But the paper lacks the separate analysis of the intrachromosomal DSB part and its difference among cell types. Figure S4 "Carbon 1034.69Kev" shows a completely flattened IMR90-ellipsoid model; why is that?
